# Elucidating protonation pathways in CO_2_ photoreduction using the kinetic isotope effect

**DOI:** 10.1038/s41467-024-44753-x

**Published:** 2024-01-10

**Authors:** Shikang Yin, Yiying Zhou, Zhonghuan Liu, Huijie Wang, Xiaoxue Zhao, Zhi Zhu, Yan Yan, Pengwei Huo

**Affiliations:** Institute of Green Chemistry and Chemical Technology, School of Chemistry and Chemical Engineering, Jiangsu University, Zhenjiang, 212013 PR China

**Keywords:** Photocatalysis, Photochemistry, Reaction kinetics and dynamics

## Abstract

The surge in anthropogenic CO_2_ emissions from fossil fuel dependence demands innovative solutions, such as artificial photosynthesis, to convert CO_2_ into value-added products. Unraveling the CO_2_ photoreduction mechanism at the molecular level is vital for developing high-performance photocatalysts. Here we show kinetic isotope effect evidence for the contested protonation pathway for CO_2_ photoreduction on TiO_2_ nanoparticles, which challenges the long-held assumption of electron-initiated activation. Employing isotopically labeled H_2_O/D_2_O and in-situ diffuse reflectance infrared Fourier transform spectroscopy, we observe H^+^/D^+^-protonated intermediates on TiO_2_ nanoparticles and capture their inverse decay kinetic isotope effect. Our findings significantly broaden our understanding of the CO_2_ uptake mechanism in semiconductor photocatalysts.

## Abstract (web summary)

Yin et al. reveal a protonation pathway in CO2 photoreduction on TiO2, challenging electron activation theories and diversifying photocatalyst design, distinct from Fischer-Tropsch and Sabatier processes.

## Introduction

The continued dependence on fossil fuels has led to a substantial increase in anthropogenic carbon dioxide (CO_2_) emissions, culminating in deleterious environmental impacts and energy crises^1,2^. An optimal strategy for addressing these challenges involves the conversion of CO_2_ into value-added products, such as CO and CH_4_, through artificial photosynthesis, which directly exploits incident sunlight and water^3,4^. However, a comprehensive understanding of the complex CO_2_ photoreduction reaction at the molecular level, particularly at the CO_2_/H_2_O/catalyst gas-liquid-solid interface, remains elusive owing to the involvement of numerous proton-coupled electron transfer processes and potential reaction pathways with various intermediates^5–7^. Elucidating the CO_2_ reduction pathway on the semiconductor catalyst surface is crucial for designing high-performance photocatalysts^8^.

Upon light exposure, a comprehensive CO_2_ photoreduction process typically encompasses water oxidation (or organic sacrificial agents, if utilized) and CO_2_ reduction half-reactions. The water oxidation half-reaction is often regarded as analogous to the oxygen-evolving reaction (OER) in water-splitting^9,10^. The CO_2_ reduction reaction encompasses multiple step-wise proton/electron transfer processes. Identifying the rate-determining step in such multi-step chemical reactions is an arduous task, yet essential for optimizing reaction systems. For example, the classic CO_2_ + 2e^−^ + 2H^+^ → CO + H_2_O (−0.53 V vs. NHE) reaction on a semiconductor photocatalyst necessitates the enrichment and activation of CO_2_ molecules at the gas-vapor-catalyst or gas-liquid-catalyst interface, followed by a reduction reaction through a series of fundamental steps involving consecutive proton and electron transfers^11^. As a linear non-polar molecule, CO_2_ is among the most stable carbon compounds. Nevertheless, the oxygen atoms in CO_2_ can donate their lone pair of electrons to surface Lewis acid centers or be protonated by Brønsted acids^12^. The carbon atom can also accept electrons from Lewis base centers, forming carbonate-like species^13^. Moreover, the π electrons of the C=O bond can interact with electron centers, leading to bond cleavage and hybridization changes from O-sp^2^ to O-sp^3^. On the surface of the semiconductor catalyst, the adsorption configuration of CO_2_ is also notably altered and influenced by the presence of water or other molecular proton donors^14–16^. All these potential reaction configurations constitute the initial steps of CO_2_ activation.

**Fig. 1:**
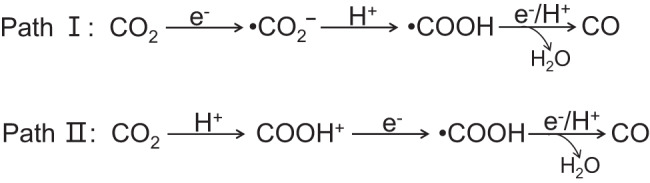
Initial CO_2_ reduction mechanism. Two feasible reaction pathways for photoreduction of CO_2_ in aqueous solution.

Figure 1 illustrates two feasible reaction pathways for CO_2_ photoreduction to CO in an aqueous solution: the electron-initiated pathway (path I) and the protonation pathway (path II). For a prolonged time, the initial step of CO_2_ activation was presumed to occur through path I, with a negatively charged CO_2_^δ•-^ species as the sole intermediate product^17,18^. However, the single-electron transfer to CO_2_ is highly endergonic due to the molecule’s negative adiabatic electron affinity^19^. Additionally, the initial CO_2_ uptake on hydrophilic surfaces of MO_*x*_/MS_*x*_ semiconductor photocatalysts is challenging, which impedes direct single-electron transfer^20,21^. Instead, a protonation pathway (path II) that first polarizes CO_2_ molecules, akin to the photocatalytic dehalogenation of non-polar halogenated aromatics^22^, appears more plausible. However, both pathways lack definitive, direct evidence for confirmation.

**Fig. 2:**
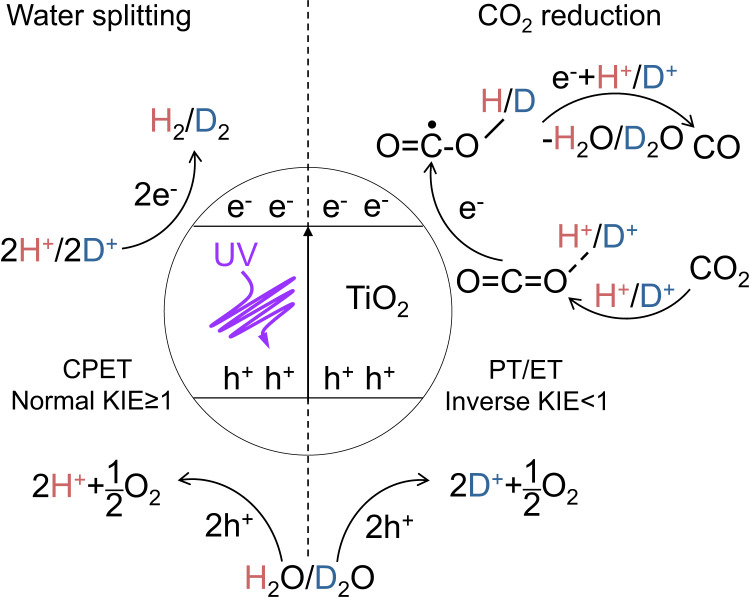
Water-splitting and CO_2_ photoreduction processes on TiO_2_. The water-splitting reaction (left) and the CO_2_ photoreduction to CO (right) with isotopically labeled H_2_O/D_2_O. CPET represents concerted proton-coupled electron transfer, PT represents proton transfer, ET represents electron transfer.

The kinetic isotope effect (KIE) is a crucial and sensitive tool for investigating reaction mechanisms by tracking the transition state of the rate-determining step using isotopically-labeled reagents^23,24^. In this study, we employed isotopically labeled H_2_O/D_2_O to determine an inverse kinetic solvent isotope effect (KSIE) of 0.2~0.9 on the photoreduction of CO_2_ to CO on TiO_2_ nanoparticles. Our findings confirm the protonation pathway with O sp^2^−hybridized O=C=O-H^+^/D^+^ intermediates (Fig. 2), providing the elucidation of the protonation pathway for CO_2_ photoreduction and shedding light on the nature of CO_2_ uptake on semiconductor photocatalysts.

## Results

### Inverse KIE of CO_2_ photoreduction

We commenced our investigation by examining the KSIE of CO_2_ photoreduction to CO in a TiO_2_/water system, employing isotopically labeled H_2_O/D_2_O, and compared it to the water-splitting reaction in analogous systems (with or without CO_2_). We first used the commercially available anatase TiO_2_ (with ~20 nm-sized nanoparticles), a prevalent photocatalyst for water-splitting and CO_2_ reduction, as a representative example of conventional metal oxide (MO_*x*_) semiconductor catalysts with hydrophilic surfaces. We quantitatively detected the reduction products (i.e., H_2_, D_2_, CO) of the water-splitting and CO_2_ photoreduction reactions through gas chromatography (Supplementary Fig. 1). Control experiments conducted without CO_2_ yielded negligible amounts of CO, suggesting that CO_2_ reduction primarily contributes to the product formation (Supplementary Fig. 2).

**Fig. 3:**
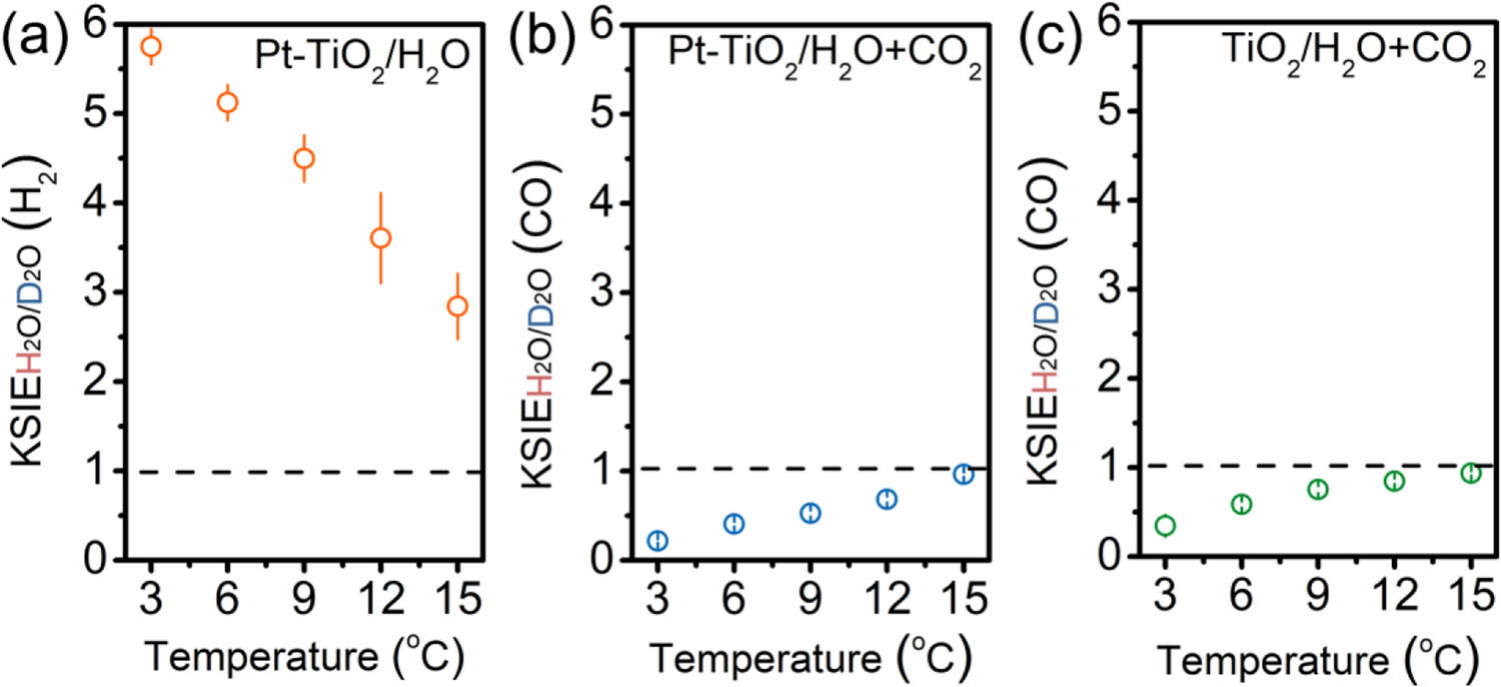
Comparison of kinetic solvent isotope effect (KSIE) in different reaction systems. **a** KSIE (H_2_) values obtained by comparing the H_2_ production kinetics of the water-splitting reaction on anatase TiO_2_ in H_2_O/D_2_O systems at different temperatures (Pt was loaded as cocatalysts, 3% chloroplatinic acid); **b** KSIE (CO) values obtained by comparing the kinetics of the CO_2_ reduction reaction on anatase TiO_2_ in H_2_O/D_2_O systems at different temperatures (Pt was loaded as cocatalysts); **c** KSIE (CO) values are given by comparing the CO production kinetics of the CO_2_ reduction reaction in the H_2_O/D_2_O systems at different temperatures without Pt cocatalysts. Error bar represents three independent experiments obtaining the standard deviation.

Figure 3a illustrates that H_2_ production from the overall water-splitting in the Pt-TiO_2_/H_2_O system (with Pt as the hydrogen evolution reaction (HER) cocatalyst) proceeds more swiftly than with D_2_O, exhibiting a normal KSIE_H2O/D2O_(H_2_) of 2.8 at 15 °C. Diminishing the reaction system’s temperature augments the KSIE value to 5.8. The same experimental phenomena could be observed regardless of whether the cocatalyst was preloaded or loaded during the reaction (Supplementary Fig. 3). This temperature-dependent KSIE is consistent with the primary KIE’s characteristics for O-H/O-D cleavage during the oxygen evolution reaction (OER), indicating direct O-H cleavage as the rate-determining step of water-splitting^25–27^. However, we observed an inverse KSIE_H2O/D2O_(CO) using the same catalyst in the presence of CO_2_ (Fig. 3b). As the temperature declined from 15 to 3 °C, the KSIE_H2O/D2O_(CO) decreased from 0.9 to 0.2. Except for adding CO_2_, all experimental conditions were congruent with the water-splitting reaction. Furthermore, the KSIE_H2O/D2O_(H_2_) under identical experimental conditions displayed >1 normal values (Supplementary Fig. 4), suggesting different rate-determining steps between CO_2_ photoreduction and water-splitting. Without Pt loading, the CO_2_ photoreduction on pristine TiO_2_ exhibited analogous inverse KSIE_H2O/D2O_(CO) values (Fig. 3c). This outcome implies that the rate-determining step encompasses hybridization changes from sp^2^ to sp^3^ in the secondary inverse KIE phenomenon, consistent with the double-bond break of O=C=O molecules instead of direct O-H cleavage in OER. By employing H_2_O/D_2_O as labeled isotopes, the observed inverse KIE denotes a configuration transition between protonated intermediates O=C=O-H^+^/D^+^ (O sp^2^) and O=C^•^−O-H/D (O sp^3^) during electron transfer, offering robust evidence for a protonation pathway involving the formation of the protonated intermediate O=C=O-H^+^ as the initial step of CO_2_ photoreduction (path II, Fig. 1). This mechanism challenges the widely accepted electron-initiated pathway (path I, Fig. 1). Note that such a protonation pathway does not rely on the presence of a water solvent. We introduced water in the form of vapor into the reaction instead of as a solvent, and the same inverse KIE could be observed (Supplementary Fig. 5). This suggests that the protonation of CO_2_ can be achieved through water vapor.

It is well-acknowledged that the characteristics of employed TiO_2_ catalysts can significantly influence their interaction with target molecules and thus lead to the change in reaction kinetics. To better ascertain whether the observed KIE changes originated from the reaction pathway itself or were influenced by the catalyst material, we conducted supplementary experiments across various TiO_2_ systems to bolster our findings. We first examined the influence of the TiO_2_ crystal structure by comparing the KIE for the CO_2_ reduction on anatase and rutile TiO_2_ (characterized by XRD and TEM/HR-TEM, see Supplementary Fig. 6 and Supplementary Fig. 7). We found that the KSIE_H2O/D2O_ (CO) on both anatase and rutile catalysts exhibited inverse KIE (<1), suggesting that the observed inverse KIE and the protonation pathway in CO_2_ reduction are common to both crystal structures. Furthermore, we examined the effect of exposed facets of the TiO_2_ catalyst. As a comparison, we synthesized anatase TiO_2_ nanosheet with high exposure of the {001} facet according to a reported method^28^, which was characterized using XRD, TEM, HR-TEM, and SAED (Supplementary Fig. 8). The KSIE_H2O/D2O_ (CO) for CO_2_ reduction on these {001}-exposed TiO_2_ nanosheets still exhibited inverse KIE < 1. These results further confirmed that the exposed facet of the TiO_2_ nanoparticles does not influence the CO_2_ reduction pathway under our experimental conditions. Finally, we evaluated the effect of oxygen defects. Oxygen vacancies on the TiO_2_ surface are often considered active sites for the oxygen evolution reaction (OER)^29^. However, their direct influence on CO_2_ reduction is less clear. We prepared oxygen-deficient TiO_2_ nanoparticles according to a reported method of NaBH_4_ calcination^30^, and characterized them using XRD, TEM, and ESR, which confirmed the presence of oxygen vacancies (Supplementary Fig. 9). The KSIE_H2O/D2O_ (CO) for CO_2_ reduction on these oxygen-deficient nanoparticles remained <1, exhibiting the secondary inverse KIE and aligning with the protonation pathway. These additional characterizations and experiments confirmed that the inverse KIE observed in the CO_2_ reduction reaction is intrinsic to the TiO_2_ material generalized to a broader range of TiO_2_-based photocatalytic systems, regardless of the crystal structure, exposed facet, or oxygen vacancy concentration.

As a conventional metal oxide semiconductor with a hydrophilic surface and moderate reduction ability, TiO_2_ demonstrates inadequate CO_2_ uptake capacity^31^. As a result, the endergonic single-electron transfer of CO_2_ → CO_2_^δ•-^ on TiO_2_ signifies a high-energy reaction. Nevertheless, in the photocatalytic dehalogenation of non-polar halogenated aromatics (e.g., polybrominated diphenyl ethers, PBDEs), a protonation pathway involving initial proton adhesion on the aromatic ring of PBDE molecules before subsequent electron transfer has been substantiated^22^. Additionally, in our recent work, we uncovered a step-wise proton transfer/electron transfer (PT/ET) pathway on TiO_2_ for the single-electron/single-proton reduction of ^t^Bu_3_ArO• and TEMPO• to ^t^Bu_3_ArOH and TEMPOH^32^. These investigations support the feasibility of the protonation pathway for CO_2_ photoreduction on TiO_2_ catalysts.

### In-situ DRIFTS measurements

To investigate the protonation pathway and monitor O=C=O-H^+^/D^+^ intermediates during photocatalytic CO_2_ reduction, we employed in-situ diffuse reflection infrared Fourier transform spectroscopy (DRIFTS) at the TiO_2_/H_2_O/CO_2_ (TiO_2_/D_2_O/CO_2_) interface. The experiment was carried out under 365 nm irradiation (3 W, LED) for 15 min, with H_2_O and CO_2_ (5 mL/min) introduced into the chamber by N_2_ flow (5 mL/min) until equilibrium was reached. We used the pre-reaction equilibrium system as a blank background and observed negative or positive IR signals during the reaction, indicating the loss or gain of species at the TiO_2_/H_2_O/CO_2_ (TiO_2_/D_2_O/CO_2_) interface. Control experiments demonstrated that in the absence of incident light, the reaction did not occur (Supplementary Fig. 10).

**Fig. 4:**
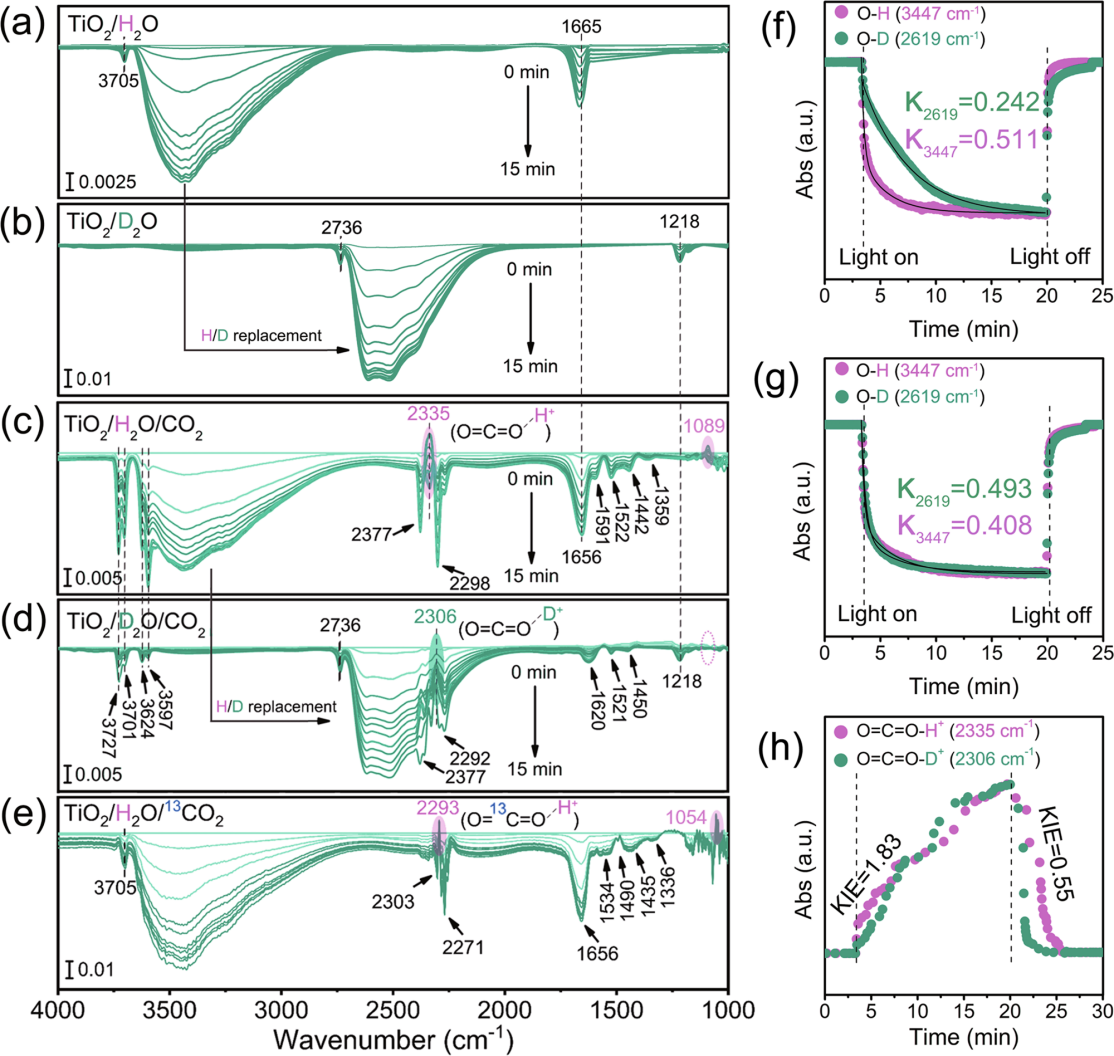
In-situ diffuse reflectance infrared Fourier transform spectroscopy (DRIFTS) measurements. DRIFTS spectra collected at the TiO_2_/H_2_O (**a**), TiO_2_/D_2_O (**b**), TiO_2_/H_2_O/CO_2_ (**c**), TiO_2_/D_2_O/CO_2_ (**d**) and TiO_2_/H_2_O/^13^CO_2_ (**e**) interfaces under constant 365 nm (3 W, LED) irradiation in 15 min; **f** Time profiles of IR signals at 3447 cm^−1^ in (**a**) and 2619 cm^−1^ in (**b**) from light-on to light-off, representing the decay kinetics of O-H and O-D in water-splitting; **g** Time profiles of IR signals at 3447 cm^−1^ in (**c**) and 2619 cm^−1^ in (**d**) from light-on to light-off, representing the decay kinetics of O-H and O-D in CO_2_ photoreduction; **h** Time profiles of IR signals at 2335 cm^−1^ and 2306 cm^−1^ (after baseline corrections to maintain positive values) from light-on to light-off, representing the formation and decay kinetics of O=C=O-H^+^ and O=C=O-D^+^. An inverse KIE was obtained during the decay process after light-off. Pink shading represents the peak position of the COOH^+^ intermediate, and green shading represents the peak position of the COOD^+^ intermediate.

Figure 4a reveals negative peaks at 3700–2800 cm^−1^ and 1665 cm^−1^ at the TiO_2_/H_2_O interface upon constant irradiation, corresponding to the O-H stretching and H-O-H bending vibrations of H_2_O molecules^33^, respectively. The weak signal at 3705 cm^−1^ represented the terminal O-H group on the TiO_2_ surface^34^. When H_2_O was replaced with D_2_O, noticeable redshifts of both O-D stretching and D-O-D bending vibrations to 2700–2100 cm^−1^ and 1218 cm^−1^ were observed (Fig. 4b), in line with the theoretical H/D replacement effect^35,36^. The decay kinetics of O-H/O-D stretching vibrations showed that the O-H signal decays much faster than the O-D signal, resulting in a direct KIE of 2.11 (Fig. 4e), consistent with the measured normal KSIE_H2O/D2O_(H_2_) values and representing features of the direct O-H/-D cleavage during overall water-splitting.

In the TiO_2_/H_2_O/CO_2_ system (Fig. 4c), negative peaks at 2377 cm^−1^ and 2298 cm^−1^ corresponding to the C=O stretching vibrations of CO_2_ were observed, along with an emerging positive signal peak at 2335 cm^−1^ adjacent to the decayed stretching vibration signals of CO_2_, likely due to the formation of the protonated CO_2_ intermediate (O=C=O-H^+^). The adhesion of a proton to the oxygen atom would alter the C=O bond and alter the effective mass of oxygen, thereby changing the vibration frequency. In addition, according to Hooke’s Law, the adhesion of protons to the oxygen nucleus in C=O bonds increases the effective mass of the oxygen atom, which subsequently results in a change in the frequency of the stretching vibrations of the C=O bond^37^. Moreover, the increasing positive signal at 1089 cm^−1^ is likely from the C=O-H^+^ bending vibration. Negative peaks at 3727 cm^−1^, 3701 cm^−1^, 3624 cm^−1^, and 3597 cm^−1^ corresponded to the weak overtone region of CO_2_ molecules^38^, and signals at 1591 cm^−1^, 1522 cm^−1^, 1442 cm^−1^, and 1359 cm^−1^ were assigned to -COOH* species, monodentate carbonate (m-CO_2-3_) groups, as well as the antisymmetric and symmetric stretching bands of bidentate carbonate (b-CO_2-3_) groups^39,40^, respectively.

To verify that the observed changes in CO_2_ FT-IR signals resulted from a surface reaction rather than a modification in the surface adsorption configuration of CO_2_ under incident light, we carried out a control experiment. This involved first running the reaction for a specified time under light, followed by the removal of the gas phase using a N_2_ flow. By subtracting the equilibrium background in N_2_ prior to the experiment, we were able to observe changes in surface-adsorbed species over time. Given that the removal of the CO_2_ gas phase would cut off the replenishment of surface CO_2_, a fading CO_2_ signal would suggest that the observed signals stemmed from the reaction rather than adsorption. Otherwise, we would observe unchanged, stable adsorbate signals. As illustrated in Supplementary Fig. 11c, d, after the abrupt removal of CO_2_, both the negative and positive signals of C=O vibration from CO_2_ species around 2330 cm^−1^ to 2340 cm^−1^ continued to decrease over time and vanished within tens of seconds. This suggests that the observed CO_2_ signals are not from a stable adsorbate but from a surface reaction. Furthermore, to validate the assignment of the protonated O=C=O-H^+^ intermediate, we replaced H_2_O with deuterated-labeled D_2_O under identical conditions. The diagnostic signal peak of the protonated intermediate shifted towards a lower wavenumber from 2335 cm^−1^ to 2306 cm^−1^ upon replacing O=C=O-H^+^ with O=C=O-D^+^ (Fig. 4d). The negative signal peaks (both stretching bands and overtone region) of CO_2_ molecules remained unchanged. This H/D replacement effect on the C=O stretching vibration of O=C=O-H^+^/D^+^ intermediates is consistent with the results of Hooke’s Law (detailed calculation formula see supplementary methods). However, the C=O-D^+^ bending vibration was not observed in O=C=O-D^+^, which likely shifts a lower frequency, beyond our in-situ DRIFTS detection range (Fig. 4d). Together with the H/D replacement experiments without CO_2_, the shift of the diagnostic peak of O=C=O-D^+^ compared to that of the unlabeled O=C=O-H^+^ provides direct evidence for the formation of protonated O=C=O-H^+^ intermediates during the CO_2_ photoreduction process at the TiO_2_/H_2_O/CO_2_ interface. Furthermore, we have an additional DRIFTS experiment using ^13^C-labeled ^13^CO_2_. As depicted in Fig. 4e, a distinct redshift from 2335 cm^−1^ to 2293 cm^−1^ of the C=O stretching vibration was observed when employing ^13^CO_2_, corresponding to the shift of the ^13^C=O stretching vibration signal in O=^13^C=O-H^+^ compared to the unlabeled ^12^C=O in O=C=O-H^+^/D^+^ (2335 cm^−1^/2306 cm^−1^) due to the ^12^C/^13^C isotope replacement effect. Moreover, the bending vibration of C=O-H^+^ at 1089 cm^−1^ was also shifted to 1054 cm^−1^ in the ^13^CO_2_ system corresponding to ^13^C=O-H^+^. These findings are highly consistent with our KIE experimental results and further validates our assignment.

### Quantum chemical calculations

We further conducted quantum chemical calculations to simulate the infrared signals of the H^+^/D^+^ protons adhered to the oxygen atom in CO_2_. The results are consistent with our assumption that the C=O stretching vibration in CO_2_ does not form a C-O-H sp^3^ structure after adhering to a H^+^/D^+^ proton, thereby a C-O signal does not appear (Supplementary Fig. 12). It remains at 2300–2400 cm^−1^ (the discrepancy between the calculation and actual data should come from different adsorption interfaces; the calculation only simulates the situation in a vacuum). The vibration frequency changes from protonated species and pristine CO_2_ due to the influence of bond energy and the effective mass of oxygen. Moreover, replacing H^+^ with D^+^ indeed causes the simulated C=O stretching vibration to shift to a lower frequency (2403 cm^−1^ → 2394 cm^−1^). Interestingly, quantum calculations also reveal possible O-H/O-D stretching vibrations (3406 cm^−1^/2490 cm^−1^), which are not clearly observed in the actual experiment due to the significant influence of water signals. More importantly, we found that the 960 cm^−1^ in O=C=O-H^+^ corresponds to the bending vibration of C=O-H^+^, which correspond to the positive signal at 1089 cm^−1^ observed in in-situ DRIFTS. In O=C=O-D^+^, the bending vibration of C=O-D^+^ shifts to a lower frequency, beyond our in-situ DRIFTS detection range, fully consistent with our observation. However, when ^13^C is used for simulation, the bending vibration of ^13^C=O-H^+^ can be seen to shift from 960 cm^−1^ to 952 cm^−1^. In our actual in-situ DRIFTS, when using ^13^CO_2_, we indeed observed a shift towards a lower wavenumber of the ^13^C=O-H+ bending vibration (1054 cm^−1^) from C=O-H^+^ (1089 cm^−1^) using unlabeled CO_2_ (Fig. 4e). This result fully support our assignment of the O=C=O-H^+^ signal.

## Discussion

In prior research, the generation of CO_2_^δ•-^ anion radicals have been detected during the photocatalytic degradation of formate on TiO_2_ nanoparticles using infrared (IR) and electron spin resonance (ESR) spectroscopy^41,42^. Although the CO_2_^δ•-^ anion radical is often cited as the exclusive intermediate of the initial step in CO_2_ photoreduction, no definitive evidence has been provided for its presence in CO_2_ photoreduction systems. On a polar TiO_2_ surface surrounded by H_2_O molecules, chemisorbed species, mainly OH^−^, produce distinct π or δ resonances, while physisorbed species have weak signals^43^. This limits the opportunities for single-electron transfer by neutral physisorbed CO_2_ molecules, which are scarce at the polar H_2_O/TiO_2_ interface. Instead, an ionized CO_2_ moiety promotes interfacial CO_2_ uptake^44^, facilitating subsequent electron/proton transfer. Under our experimental conditions, the only visible positive signal peak after light illumination corresponds to the protonated O=C=O-H^+^/D^+^ signal. This finding contradicts previous understandings of the CO_2_ photoreduction mechanism and suggests a protonation pathway^45^.

**Fig. 5:**
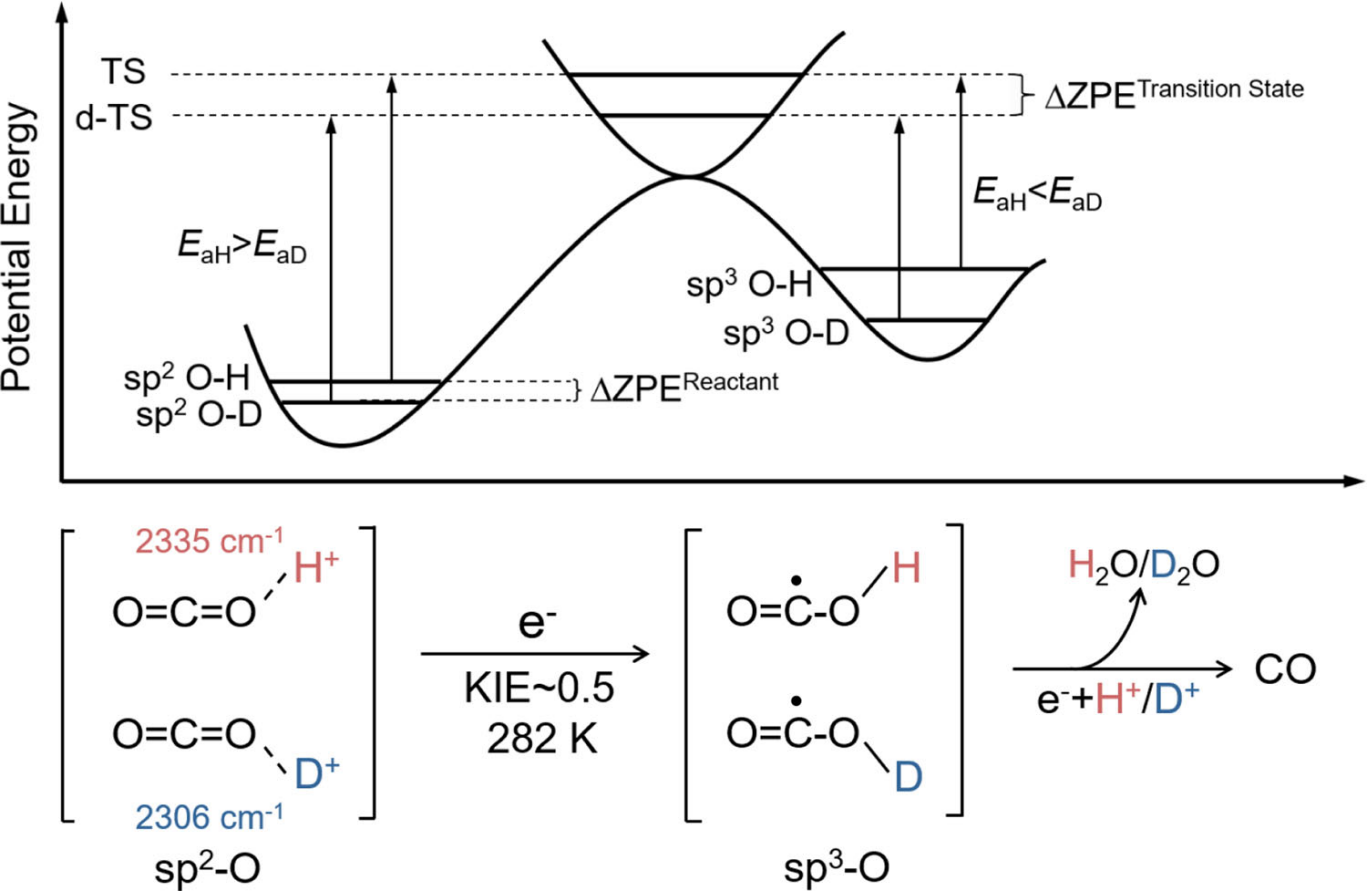
The source of inverse KIE. Schematic illustrations and energetic profiles of the O=C=O-H^+^/D^+^ → O=C^**•**^−O-H/D electron transfer process. TS represents transition state, ZPE represents zero-point energy.

We also compared the decay kinetics of O=C=O-H^+^/D^+^ and O-H/O-D during the reaction. The inverse kinetic isotope effect (KIE) of the O sp^2^ → O sp^3^ hybrid transition process is a classic phenomenon in reaction kinetics associated with the disparity in the vibration frequency of chemical bonds^22,46^. The observed inverse KIE of O=C=O-H^+^/D^+^ decay (KIE = 0.55) provides strong evidence for the protonation pathway (Fig. 4h; Supplementary Fig. 13), which involves the O=C=O-H^+^/D^+^ → O=C^•^−O-H/D double-bond break with a hybridization change from O sp^2^ → O sp^3^ via additional electron transfer (Fig. 5). In most reactions, the overall rate is determined by the slowest step, known as the rate-determining step^47^. In our system, without CO_2_, the direct breakage of the O-H/O-D bond undeniably constitutes the rate-determining step, hence its KIE is greater than 1 (Fig. 4f). However, the decay kinetics of O-H/O-D stretching vibration also exhibited an inverse KIE = 0.827 in the presence of CO_2_ (Fig. 4g), indicating that the slower reduction reaction of CO_2_ (in this case, the reduction of the protonated intermediate) becomes the rate-determining step.

In this study, we unveil a mechanism governing the photoreduction of CO_2_ on semiconductor catalysts, which transpires via a protonation pathway. We report the formation of an O=C=O-H^+^ intermediate, which exhibits an inverse KIE during the subsequent electron transfer process. This electron transfer process prompts the conversion of the sp^2^−hybridized O=C=O-H^+^/D^+^ species into the sp^3^−hybridized O=C^•^−O-H/D species. Utilizing isotopically labeled in-situ DRIFTS, we successfully discern the formation of H^+^/D^+^-protonated O=C=O-H^+^/D^+^ intermediates on TiO_2_ nanoparticles and capture their inverse decay KIE. This research substantially broadens our comprehension of the CO_2_ uptake mechanism in semiconductor photocatalysts, necessitating a re-examination of long-held assumptions within the field. Our findings hold significant potential for advancing the development of more efficient and sustainable photocatalytic CO_2_ reduction technologies in the future.

## Methods

### Materials

Commercial titanium dioxide (TiO_2_, anatase, 20 nm), sodium borohydride (NaBH_4_), tetrabutyl titanate, hydrofluoric acid (HF, 40 wt%), chloroplatinic acid (H_2_PtCl_6_·6H_2_O), ethanol and deuterium oxide (D_2_O, 99.9 atom % D) were purchased from Shanghai McLean Biochemical Technology Co., Ltd. All reagents used in the synthesis were analytically pure and had not been further purified. Deionized water was obtained from a purified distillation unit in the laboratory. Before any photocatalytic reaction experiments, TiO_2_ samples were first calcinated and then illuminated by an ultraviolet lamp (365 nm, 160 mW∙cm^−2^) in water.

### Synthesis of (001) exposed TiO_2_ nanosheet

In a typical synthesis, 12.5 mL of tetrabutyl titanate was mixed with 2 mL of HF solution, under stirring for 30 min. The solution was then transferred into a 50-mL Teflon-lined autoclave, and kept at 180 °C for 24 h. After the solvothermal reaction, the resulting white precipitates were collected and washed with ethanol and distilled water for three times. The samples were dried in a vacuum oven at 60 °C for 12 h.

### Synthesis of oxygen-deficient TiO_2_

1 g TiO_2_ nanoparticle powder was mixed with 2 g NaBH_4_ and the mixture was ground for 30 min thoroughly. Then the mixture was transferred into a porcelain boat, and placed in a tubular furnace, heated from room temperature to 350 °C/1 h under an Ar atmosphere at a heating rate of 10 °C min^−1^. After naturally cooling down to room temperature, the colored TiO_2_ was obtained, simply washed with deionized water and ethanol several times to remove unreacted NaBH_4_, and dried at 70 °C.

### Water-splitting experiments

In a typical procedure, 50 mg TiO_2_ powder was dispersed in 10 mL deionized water (H_2_O) and 10 mL deuterium water (D_2_O), respectively. Next, 3 wt% Pt as cocatalysts was loaded via in-situ photo deposition using H_2_PtCl_6_·6H_2_O without any sacrificial agents. After irradiation with an ultraviolet lamp (365 nm, 160 mW∙cm^−2^), Gas products were determined by using a gas chromatography (GC-7900) equipped with the TCD thermal conductivity detector and the carrier gas was chosen Ar.

### CO_2_ photoreduction experiments

CO_2_ photoreduction was carried out in a sealed self-made 150 mL stainless-steel reactor with an ultraviolet lamp (365 nm, 160 mW∙cm^−2^) as the light source. In a typical procedure, 50 mg catalyst was dispersed in 10 mL deionized water (H_2_O) and 10 mL deuterium water (D_2_O), respectively. CO_2_ was then introduced into the reactor and bubbled for 25 min to completely remove air. Gas products were detected by the gas chromatography (GC-7920, China) equipped with hydrogen flame ionization detector (FID) and thermal conductivity detector (TCD). In addition, the control experiment had the same experimental conditions as described above except for the addition of 3 wt% Pt as cocatalysts; In the gas-solid reaction system, 50 mg catalyst was dispersed in quartz grooves, add 2 ml of water or deuterated water to the bottom of the 150 ml reactor with no direct contact with the catalyst, assuring that water participates in the reaction in vapor state. CO_2_ flow was then introduced into the reactor for 25 min before light-on.

### In-situ DRIFTS experiments

In-situ diffuse reflection infrared Fourier transform spectroscopy (DRIFTS) experiments were conducted on a Nicolet iS10 (Thermo) machine according to our previous work^47^. In a typical procedure, catalyst sample was sealed in the reaction chamber with a quartz window. CO_2_ and H_2_O (or D_2_O) were carried into the reaction chamber by N_2_ flow until equilibrium. After taking the equilibrium system before reaction as the blank background, IR signals were collected in-situ during the incident irradiation of a 365 nm LED lamb (3 W) through the quartz glass window.

### Hooke’s law

Taking diatomic as an example, when the diatomic is telescopic and vibrating, they can be approximated as a simple harmonic oscillator. Given two bodies, one with mass m_1_ and the other with mass m_2_, the equivalent one-body problem, with the position of one body with respect to the other as the unknown, is that of a single body of mass; where the equivalent mass of O=C=O-H^+^ is m_1_ = 12 (C), m_2_ = 17 (O-H, *v*_1_ = 2335 cm^−1^); The equivalent mass of O=C=O-D^+^ is m_1_ = 12 (C), m_2_ = 18 (O-D, *v*_2_ = 2306 cm^−1^).

1 $${{{{{\rm{Composite\; mass}}}}}}\!:{{{{{\rm{\mu }}}}}}=\frac{1}{\frac{1}{{{{{{{\rm{m}}}}}}}_{1}}+\frac{1}{{{{{{{\rm{m}}}}}}}_{2}}}=\frac{{{{{{{\rm{m}}}}}}}_{1}{{{{{{\rm{m}}}}}}}_{2}}{{{{{{{\rm{m}}}}}}}_{1}+{{{{{{\rm{m}}}}}}}_{2}}$$

2 $${{{{{\rm{Vibration\; frequency}}}}}}\!:{{{{{\rm{\upsilon }}}}}}=\frac{1}{2{{{{{\rm{\pi }}}}}}}\sqrt{\frac{{{{{{\rm{k}}}}}}}{{{{{{\rm{\mu }}}}}}}}$$

When *v*_1_ = 2335 cm^−1^:

The force constants of chemical bonds:

$${{{{{\rm{k}}}}}}={{{{{\rm{\mu }}}}}}{\left({2{{{{{\rm{\pi }}}}}}{{{{{\rm{\upsilon }}}}}}}_{1}\right)}^{2}=\frac{{{{{{{\rm{m}}}}}}}_{1}{{{{{{\rm{m}}}}}}}_{2}}{{{{{{{\rm{m}}}}}}}_{1}+{{{{{{\rm{m}}}}}}}_{2}}{\left({2{{{{{\rm{\pi }}}}}}{{{{{\rm{\upsilon }}}}}}}_{1}\right)}^{2}=\frac{12\times 17}{12+17}{\left(2\times 3.14\times 2335\right)}^{2}=1.51\times {10}^{9}$$

When the equivalent mass of O=C=O-D^+^ is m_1_ = 12, m_2_ = 18:

$${{{{{{\rm{\upsilon }}}}}}}_{2}=\frac{1}{2{{{{{\rm{\pi }}}}}}}\sqrt{\frac{{{{{{\rm{k}}}}}}}{{{{{{\rm{\mu }}}}}}}}=\frac{1}{2{{{{{\rm{\pi }}}}}}}\sqrt{\frac{{{{{{\rm{k}}}}}}}{\frac{{{{{{{\rm{m}}}}}}}_{1}{{{{{{\rm{m}}}}}}}_{2}}{{{{{{{\rm{m}}}}}}}_{1}+{{{{{{\rm{m}}}}}}}_{2}}}=}\frac{1}{2\times 3.14}\sqrt{\frac{1.51\times {10}^{9}}{\frac{12\times 18}{12+18}}=2308}$$

### Supplementary information

Supplementary Information

Peer Review File

### Source data

Source Data

## Data Availability

The data supporting the findings of this study are available within the article and its Supplementary Information files. All other relevant source data are available from the corresponding author upon request. Source data are provided with this paper.
