# Supplementary material for: Elucidating protonation pathways in CO2 photoreduction using the kinetic isotope effect
Source: Nat Commun. 2024 Jan 10;15:437. doi: 10.1038/s41467-024-44753-x (PMC10781958; doi:10.1038/s41467-024-44753-x)
Supplement: Supplementary file 1 — Supplementary Information [file 41467_2024_44753_MOESM1_ESM.pdf]

Supplementary Information

Elucidating protonation pathways in CO_2 photoreduction using the kinetic isotope effect

Shikang Yin[1], Yiying Zhou[1], Zhonghuan Liu[1], Huijie Wang[1], Xiaoxue Zhao[1], Zhi Zhu[1], Yan Yan[1]*, Pengwei Huo[1]*

[1]Institute of Green Chemistry and Chemical Technology, School of Chemistry and Chemical Engineering, Jiangsu University, Zhenjiang 212013, PR China, E-mail: dgy5212004@163.com and huopw@ujs.edu.cn

Supplementary Figures

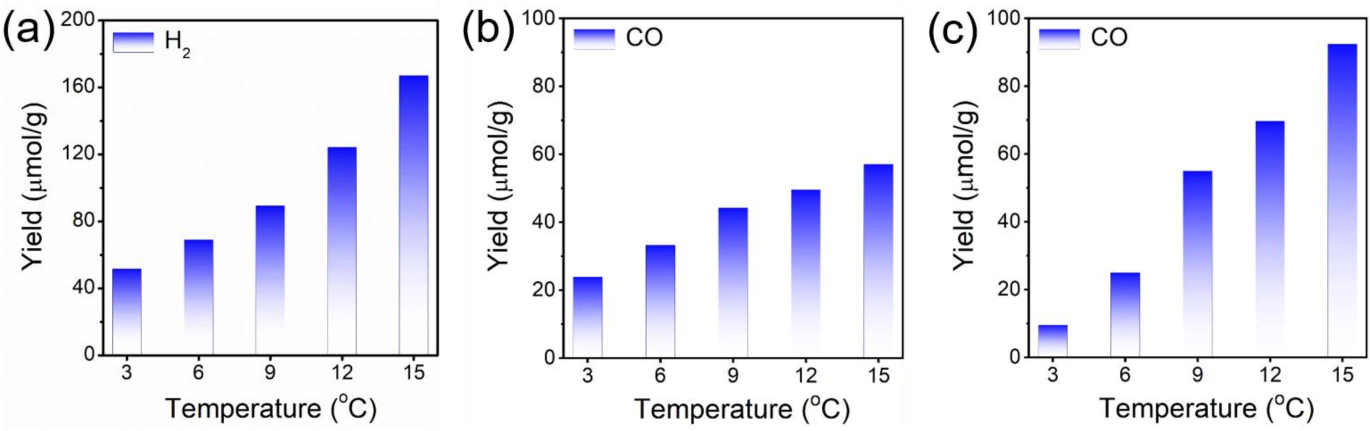

Supplementary Figure 1. Comparison of the water-splitting and CO_2 reduction performances. (a) The H_2 evolution for the water-splitting reaction run in 5 h using the TiO_2 photocatalyst at different temperatures (Pt was loaded as co-catalysts); (b) The CO evolution for the CO_2 reduction reaction run in 5 h using the TiO_2 photocatalyst at different temperatures (without Pt co-catalysts); (c) The CO evolution for the CO_2 reduction reaction run in 5 h using the Pt/TiO_2 photocatalyst at different temperatures (Pt was loaded as co-catalysts).

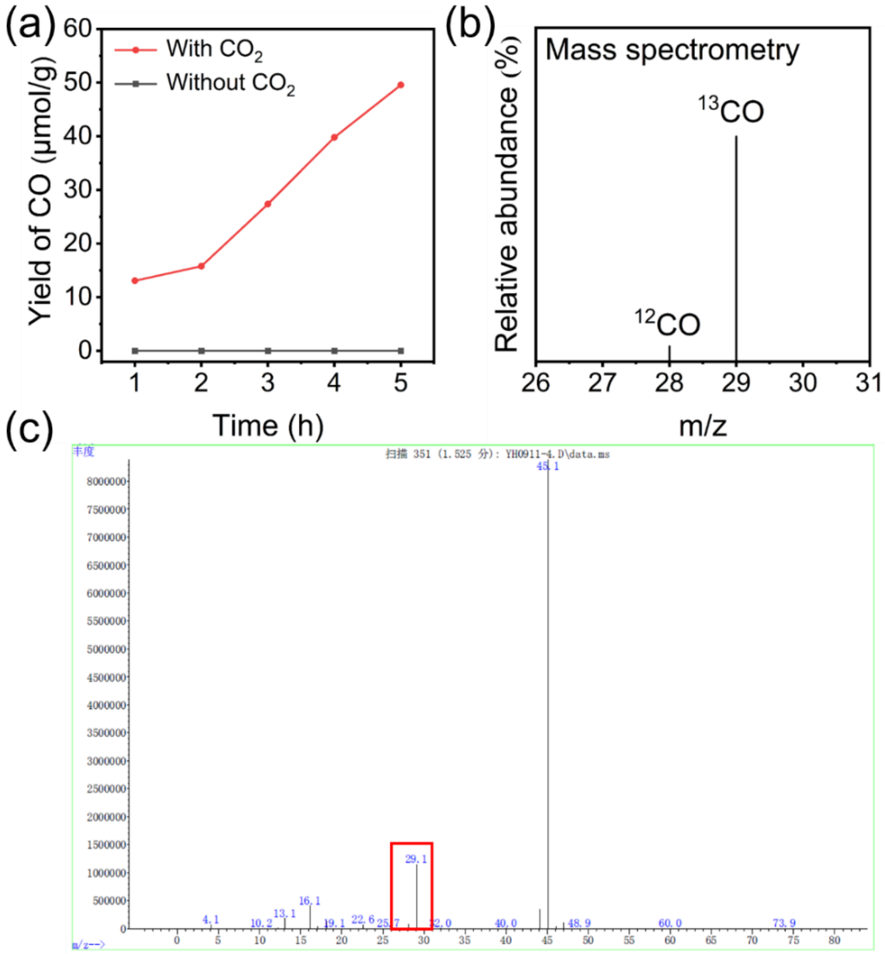

Supplementary Figure 2. Characterization of sample performance and mass spectrometric. (a) The comparison of CO

production rate with/wothout CO_2 on anatase TiO_2 catalyst (b) Mass spectrometry analyses of ^{13}CO (m/z =29) when using $^{13}CO_2$ in the CO_2 photoreduction on the TiO_2 catalyst. (c) raw data report of the mass spectrum.

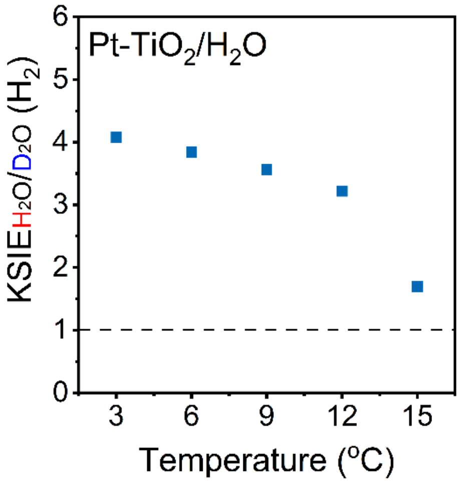

Supplementary Figure 3. Control experiments show water-splitting KIE. KSIE (H_2) values obtained by comparing the H_2 production kinetics of the water-splitting reaction in the H_2O/D_2O systems at different temperatures (Pt was pre-deposited to exclude the influence of chloride ions).

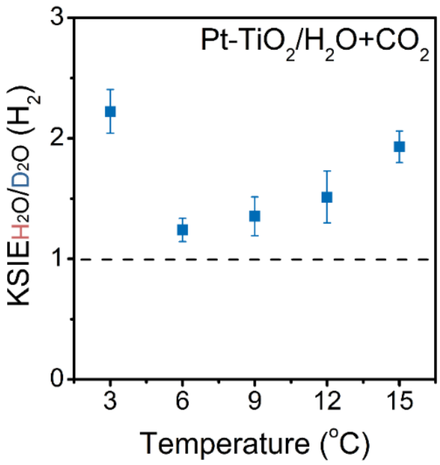

Supplementary Figure 4. Control experiments show CO_2 reduction KIE. KSIE (H_2) values are given by comparing the kinetics of the CO_2 reduction reaction in the H/D system at different temperatures (Pt was loaded as co-catalysts, 3% chloroplatinic acid). Since the produced hydrogen will continue to participate in the subsequent CO_2 reduction process, its kinetic curve is greatly affected by the CO_2 reduction reaction, but it still shows a KSIE greater than 1 under different temperature conditions.

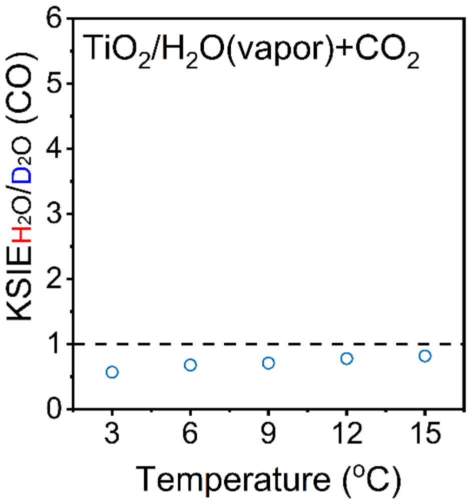

Supplementary Figure 5. Control experiments show the effect of water vapor. KSIE (CO) values are given by comparing the kinetics of the CO_2 reduction reaction on anatase TiO_2 catalyst with H_2O/D_2O in the vapor state at different temperatures.

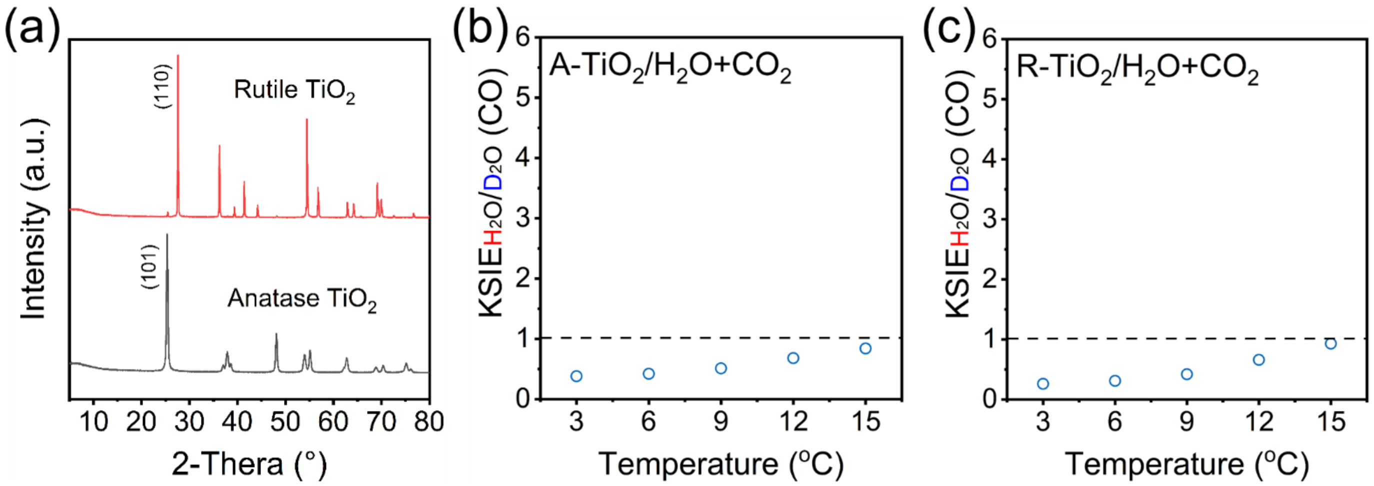

Supplementary Figure 6. Comparison of sample crystalline structure and KIE. (a) XRD patterns of rutile and anatase TiO_2; (b, c) KSIE (CO) values on anatase and rutile TiO_2 catalysts in the H_2O/D_2O systems at different temperatures.

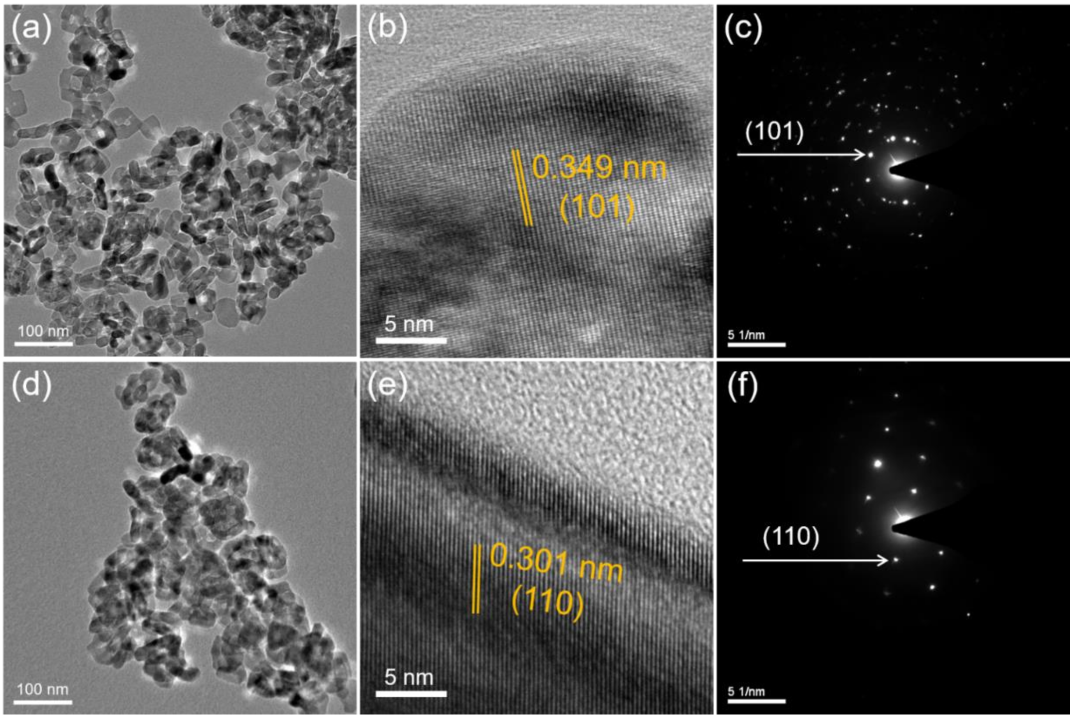

Supplementary Figure 7. Comparison of sample morphologies. (a, b, c) TEM/HR-TEM images and SAED pattern of the anatase TiO_2 catalyst; (d, e, f) TEM/HR-TEM images and SAED pattern of the rutile TiO_2 catalyst.

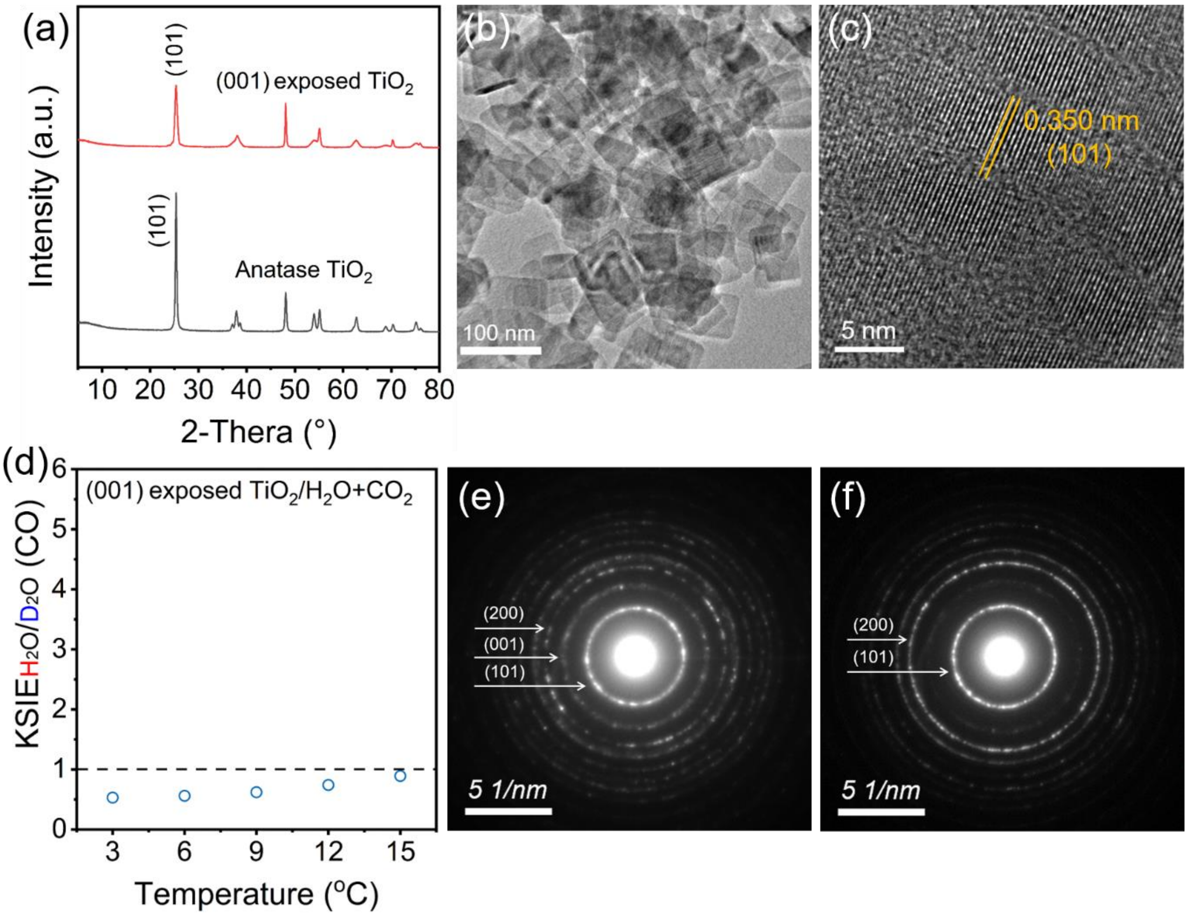

Supplementary Figure 8. Comparison of sample crystal planes. (a) XRD patterns of the (001) facet exposed TiO_2 nanosheet catalyst and the normal anatase TiO_2 catalyst; (b, c) TEM and HR-TEM images of the (001) exposed TiO_2 nanosheet; (d) KSIE (CO) values on {001} facet exposed TiO_2 nanosheet catalyst in the H_2O/D_2O systems at different temperatures; (e) SAED pattern of the normal anatase TiO_2 catalyst; (f) SAED pattern of the the (001) facet exposed TiO_2 catalyst.

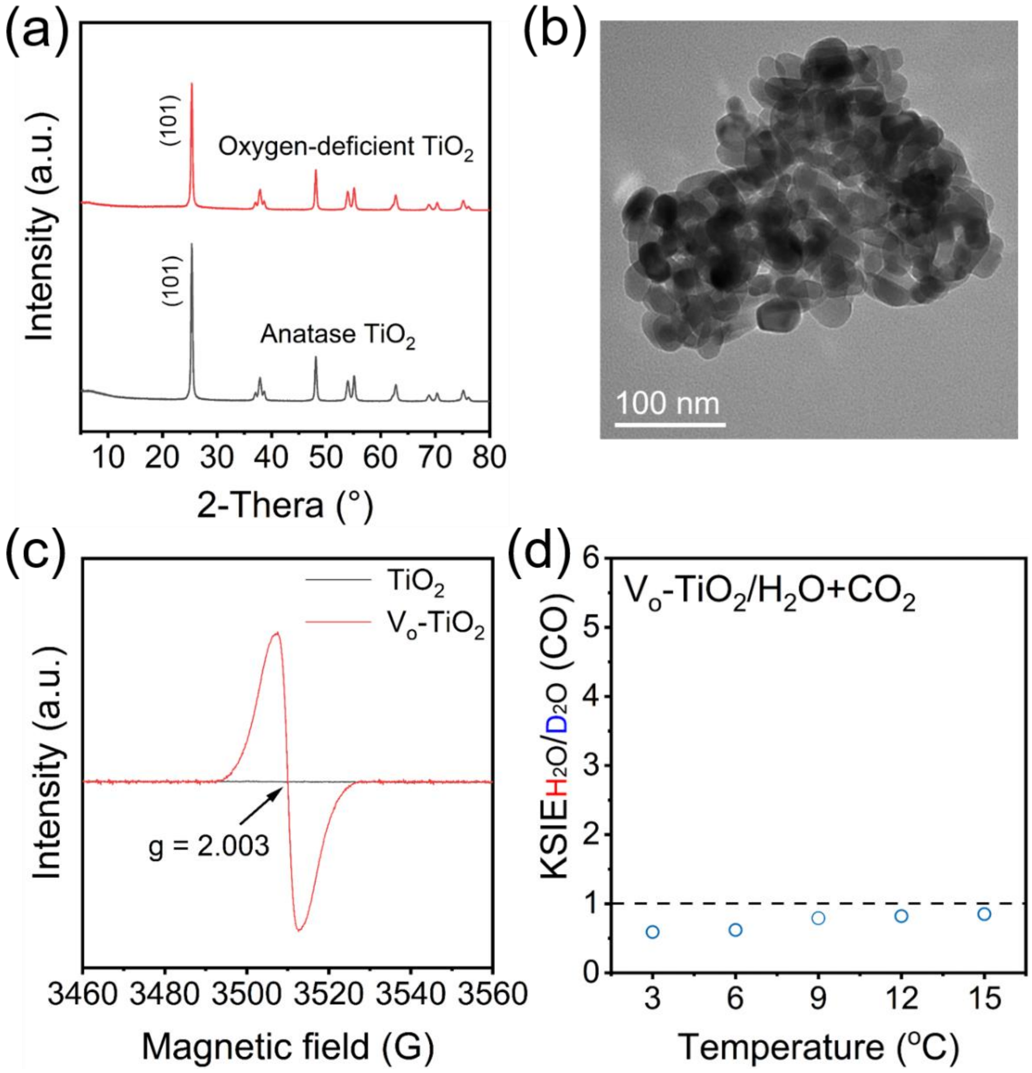

Supplementary Figure 9. Control experiments show the effect of oxygen-deficient. (a) XRD patterns of oxygen-deficient TiO_2 (Vo-TiO_2) and normal anatase TiO_2 catalysts; (b) TEM image of the oxygen-deficient TiO_2 (Vo-TiO_2) catalyst; (c) EPR spectra of oxygen-deficient TiO_2 (Vo-TiO_2) and normal anatase TiO_2 catalysts; (d) KSIE (CO) values of the CO_2 reduction reaction on the oxygen-deficient TiO_2 catalyst (Vo-TiO_2) in the H_2O/D_2O systems at different temperatures.

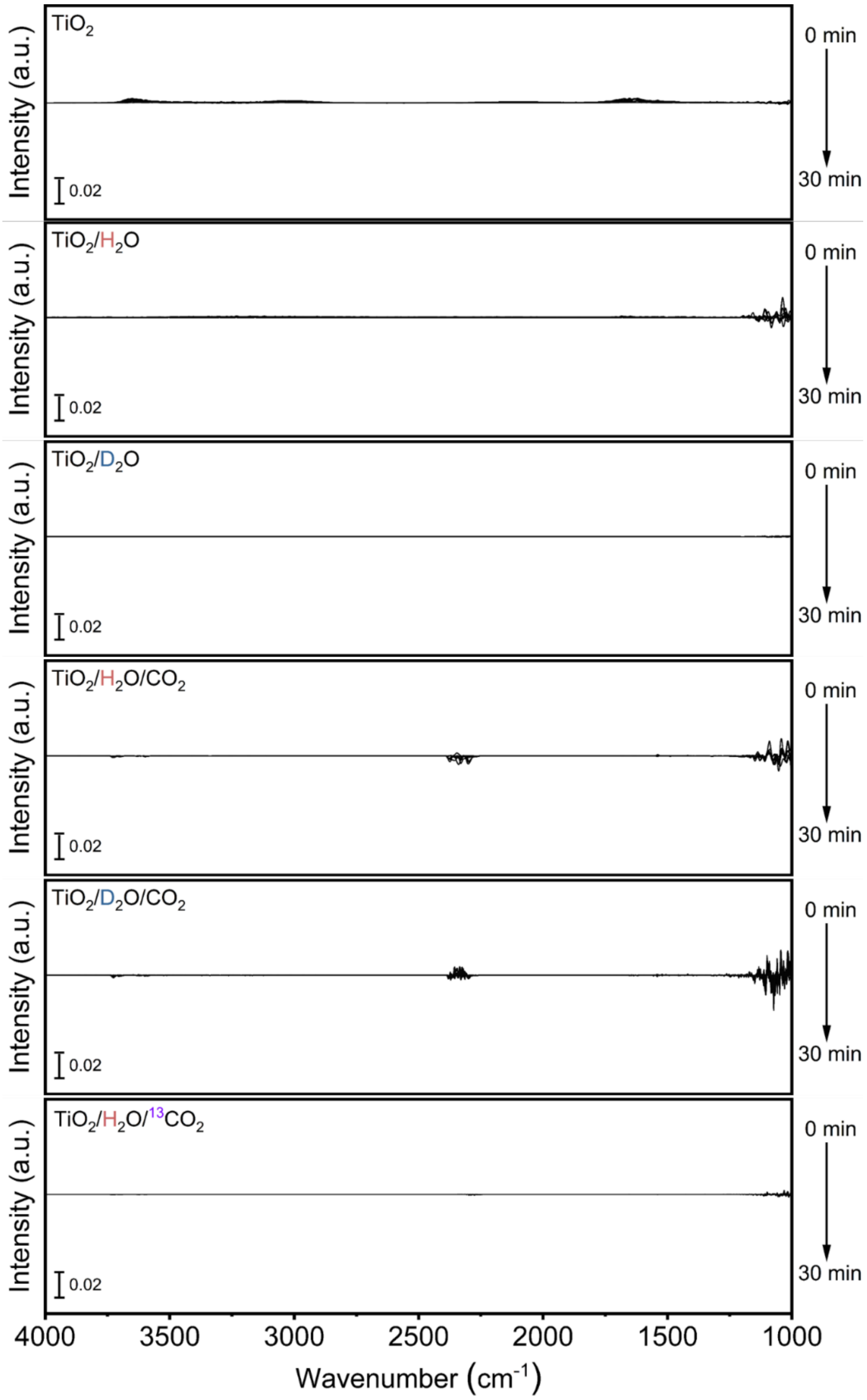

Supplementary Figure 10. Control experiments show infrared absorption spectra under dark reaction. In-situ DRIFTS spectra collected at the TiO_2, TiO_2/H_2O, TiO_2/D_2O, $TiO_2/H_2O/CO_2$, $TiO_2/D_2O/CO_2$ and $TiO_2/H_2O/^{13}CO_2$ interfaces in the dark within 30 min.

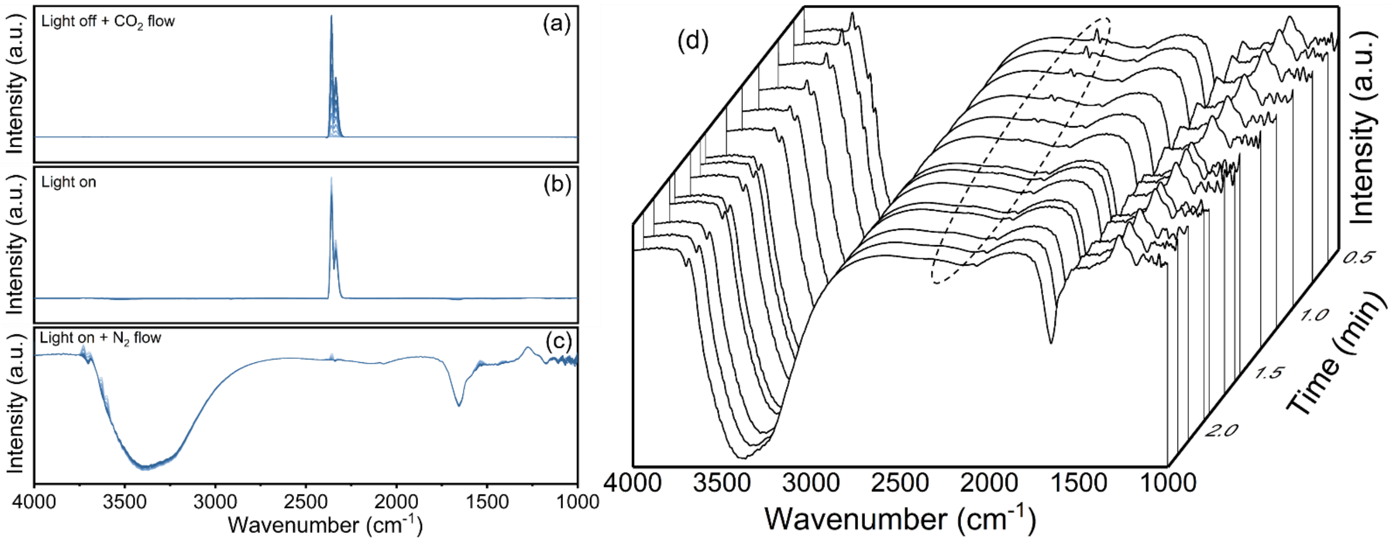

Supplementary Figure 11. Control experiments show infrared absorption spectra under different conditions. (a) In-situ DRIFTS spectra taking with the bare TiO_2 surface (in N_2 flow) as the background then CO_2 was introduced into the system in the dark for 15 min. (b) After 15 min in the dark, the identical system of (a) was then illuminated by a 365 nm (3W) LED lamb for 10 min. (c) during the continuous 365 nm (3W, LED) irradiation, an abrupt removal of the CO_2 gas-phase by the N_2 flow was conducted. (d) Waterfall profiles of the in-situ DRIFTS spectra after the abrupt CO2 gas-phase removal. Surface CO_2 species faded out in 1 min.

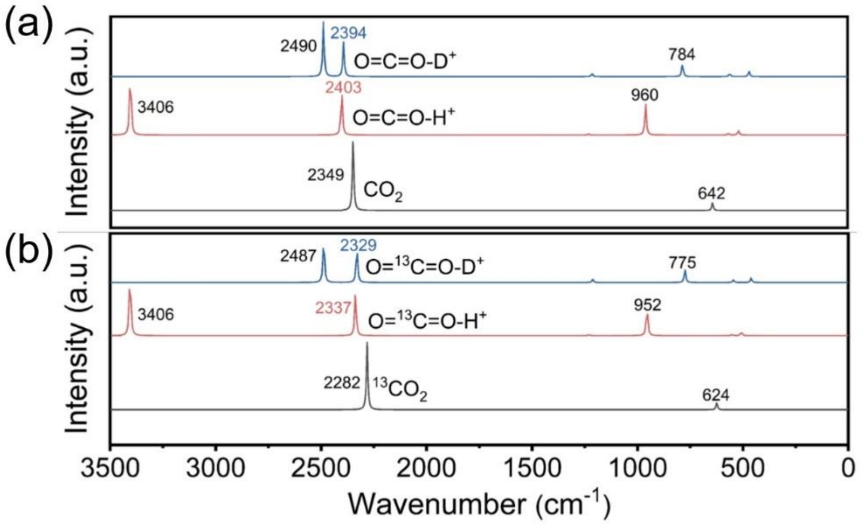

Supplementary Figure 12. Quantum chemical calculations. (a) Quantum chemical calculation of the infrared spectra of CO_2, $O{=}C{=}O\text{-}H^+$ and $O{=}C{=}O\text{-}D^+$; (b) Quantum chemical calculation of the infrared spectra of $^{13}CO_2$, $O{=}^{13}C{=}O\text{-}H^+$ and $O{=}^{13}C{=}O\text{-}D^+$.

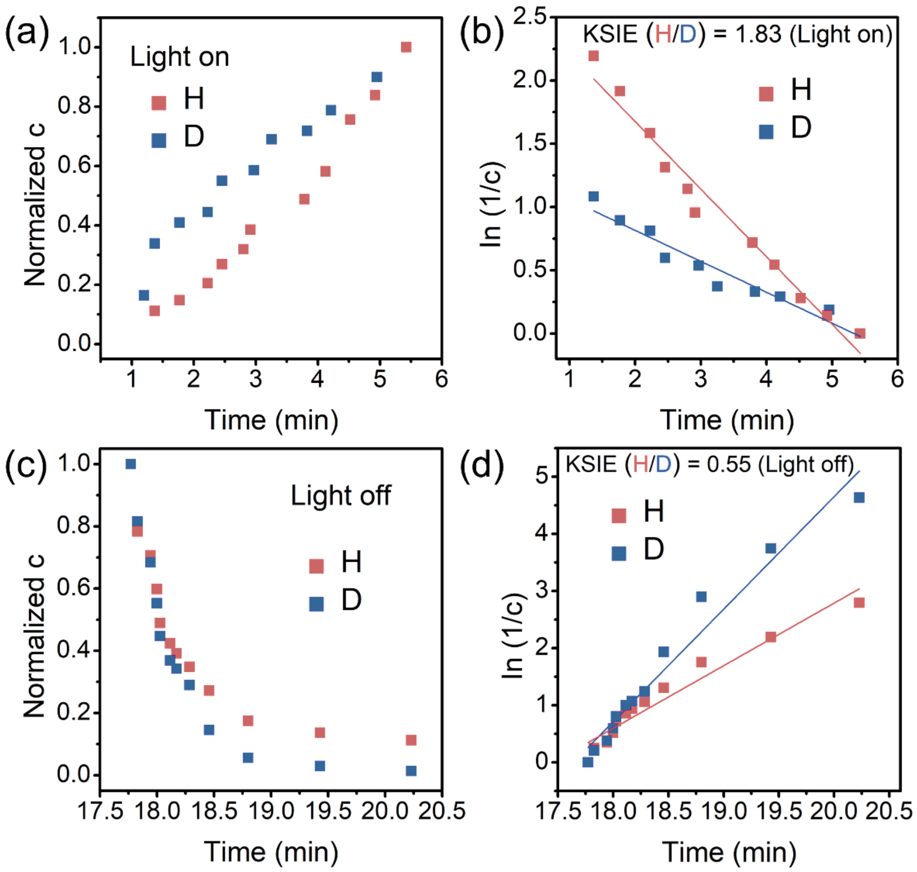

Supplementary Figure 13. Control experiments show fitted data for IR. (a, b) KSIE value was given by comparing the rising signals of protonated intermediates (O=C=O-H^+/D^+) in the H/D systems, light-on; (c, d) KSIE value was given by comparing the decay signals protonated intermediates (O=C=O-H^+/D^+) in the H/D systems, light off.

Supplementary Tables

Supplementary Table 1 Assignment of CO_2, O=C=O-H^+/D^+, O=^{13}C=O-H^+/D^+ infrared spectral vibrational modes by quantum chemical calculations.

	Wavelength (cm^{-1})	Vibration mode
CO_2	642	O=C=O bending vibration
	2349	O=C=O asymmetric stretching vibration
O=C=O-H^+	960	C=O-H bending vibration
	2403	O=C=O asymmetric stretching vibration, C-H stretching vibration
	3406	O-H stretching vibration
O=C=O-D^+	469	O=C=O bending vibration, C-D bending vibration
	561	O=C=O bending vibration
	784	C=O-D bending vibration
	1214	O=C=O symmetric stretching vibration
	2394	O=C=O asymmetric stretching vibration, C-D stretching vibration
	2490	O-D stretching vibration
$^{13}CO_2$	624	O=C=O bending vibration
	2282	O=C=O asymmetric stretching vibration
O=^{13}C=O-H^+	952	C=O-H bending vibration
	2337	O=C=O asymmetric stretching vibration, C-H stretching vibration
	3406	O-H stretching vibration
O=^{13}C=O-D^+	461	O=C=O bending vibration, C-D bending vibration
	545	O=C=O bending vibration
	775	C=O-D bending vibration
	1212	O=C=O symmetric stretching vibration
	2329	O=C=O asymmetric stretching vibration, C-D stretching vibration
	2487	O-D stretching vibration
